# Nutritional intervention improved dietary intake among patients receiving methadone maintenance treatment

**DOI:** 10.1016/j.dadr.2026.100448

**Published:** 2026-05-22

**Authors:** Dror Ben Noach, Anat Sason, Ronit Anbar, Limor Ben Haim, Miriam Adelson, Einat Peles

**Affiliations:** a Diet & Nutrition department, Tel Aviv Sourasky University Medical Center, Israel; b Department of Biochemistry, Food Science and Nutrition, Robert H. Smith Faculty of Agriculture, Food and Environment, The Hebrew University of Jerusalem, Rehovot, Israel; c Dr. Miriam and Sheldon G. Adelson Clinic for Drug Abuse, Treatment and Research, Psychiatry Division, Tel Aviv Sourasky University Medical Center, Tel Aviv, Israel; d Grey Faculty of Medical and Health Sciences, Tel Aviv University, Tel Aviv, Israel; e Sagol School of Neuroscience, Tel Aviv University, Tel Aviv, Israel

**Keywords:** Methadone maintenance treatment, Group nutritional therapy, Ultra-processed foods, Dietary quality, Opioid use disorder

## Abstract

**Objectives:**

As patients receiving methadone maintenance treatment (MMT) exhibit elevated rates of overweight, and poor dietary quality, we studied the effectiveness of a group nutritional-therapy on dietary quality, anthropometric measures, body composition, and quality of life.

**Methods:**

A randomized controlled trial among MMT patients with BMI ≥ 25 kg/m² was assessed. The intervention group (n = 42) received 10 weekly group nutritional therapy sessions, and controls (n = 28) received a single 15-minute individual nutritional session. Anthropometric measurements, body composition analysis (bio-impedance analysis), dietary intake evaluation, and quality of life (SF-36) were assessed. Data were analyzed using repeated-measures analyses.

**Results:**

Intervention and control groups had reductions in body fat percentage (p = 0.008) with comparable proportions of patients who lost weight (42.9% and 50%, respectively, p = 0.6). Water consumption increased and ultra-processed food consumption decreased in both groups. The intervention group only demonstrated caloric reduction in carbohydrate (48.2% to 44.7%) and increase in protein (13.8% to 16.9%). Physical-functioning domain of quality of life was maintained in the intervention group but declined in controls. When stratified by weight loss, scores improved among participants who lost weight in the intervention group, but declined among those who did not, as well as among all participants in the control group regardless of weight change.

**Conclusions:**

Intervention effectively improved dietary quality in MMT patients, and among those who succeeded in losing weight, it also improved quality of life (physical-functioning). The brief nutritional guidance provided to controls, and the increased dietary awareness from study participation, may have contributed to general nutritional improvements.

## Highlights

• Dietary quality among MMT patients improved following a group intervention.
• Water consumption increased and ultra-processed food consumption decreased.
• A reduction in carbohydrate proportion and increase in protein proportion were achieved.
• Quality of life improved only among patients in the intervention group who lost weight.

## 1 Introduction

Patients on methadone maintenance treatment (MMT) may experience weight gain which is associated with metabolic syndrome, as has been consistently reported across multiple studies (Vallecillo et al., 2018, Peles et al., 2016). Weight gain in patients on MMT appears to be multifactorial, stemming from several mechanisms including an increased preference for sweet foods due to neurobiological alterations in brain reward systems (Kolarzyk et al., 2005, Green et al., 2013), and the prevalent use of psychiatric medications that may contribute to weight gain (Schlienz et al., 2018).

Thus, a substantial behavioral shift toward increased food consumption following initiation of MMT is likely driven by the time and financial resources previously devoted to acquiring street drugs (Sason et al., 2018).

Patients on MMT may exhibit significantly higher rates of overweight and obesity compared to the general population. In a study of 122 patients on MMT, 29.5% were overweight and 17.2% were obese (Vallecillo et al., 2018). Another study that followed patients on MMT found a significant increase in body mass index (BMI) from 22.5 to 24.4 kg/m² over an average follow-up period of 270 days (Peles et al., 2016). This weight gain is concerning given that overweight and obesity are associated with an increased risk of cardiovascular disease, type 2 diabetes, metabolic syndrome, and increased mortality (Morgan-Bathke et al., 2023).

Body composition analysis provides important insights beyond BMI measurements, particularly with regard to metabolic health and disease risk. While BMI serves as a useful screening tool, body composition assessment allows for differentiation between fat mass and lean body mass, thereby providing a more precise evaluation of metabolic risk factors (Lin et al., 2021; Muller et al., 2012). One study examining body composition in patients on MMT reported elevated body fat percentages consistent with obesity classifications (Sadek et al., 2016).

The nutritional profile of patients on MMT is characterized by poor dietary quality, including excessive sugar consumption, often exceeding 25–30% of total caloric intake, high intake of ultra-processed foods, and inadequate consumption of vegetables and dietary fiber (Furulund et al., 2024, Nolan and Scagnelli, 2007).

Sugar consumption among patients on MMT has been documented to be substantially higher than recommended guidelines, with studies reporting sugar intake constituting 30–31% of total daily energy intake (Zador et al., 1996, Saeland et al., 2011). This dietary pattern aligns with the documented preference for sweet foods among patients on MMT, likely mediated by μ-opioid receptor activation within brain reward pathways (Mysels et al., 2010).

Ultra-processed foods (UPF), characterized by high caloric density, added sugars, unhealthy fats, and low nutritional quality, have been increasingly recognized as a major contributor to poor health outcomes (Contreras-Rodriguez et al., 2022). UPF consumption is associated with an increased risk of obesity, metabolic syndrome, type 2 diabetes, cardiovascular disease, and increased mortality (Dai et al., 2024). While previous research has established that patients on MMT tend to consume foods characteristic of the UPF dietary profile such as sweets, no studies have specifically examined UPF consumption patterns in this population (Furulund et al., 2024, Nolan and Scagnelli, 2007).

Patients on MMT also typically exhibit lower quality of life scores compared to the general population, with particular impairments in physical functioning and mental health domains (De Maeyer et al., 2010). A systematic review of 38 studies demonstrated that quality of life among individuals with OUD is significantly lower compared to both the general population and individuals with other health conditions (De Maeyer et al., 2010). Our previous randomized controlled trial investigated a nutritional intervention in this population, which consisted of two educational sessions on proper nutrition habits and found improvements in nutritional knowledge and eating behaviors but no changes in BMI (Sason et al., 2018). Currently, there are no specific nutritional guidelines for patients on MMT, despite their elevated risk for metabolic complications.

Recent review (Waddington et al., 2023) reported that patients undergoing methadone maintenance therapy exhibit irregular and nutritionally inadequate dietary intake, however, this literature is limited to small observational studies, with a notable absence of intervention-based research.

In the current study, for the first time, we comprehensively evaluated, in an intervention-controlled trial, the effects of a group nutritional intervention on anthropometric measures, body composition, dietary quality, and quality of life in patients on MMT with overweight and obesity.

The primary objective was to determine whether group nutritional therapy could improve these outcomes compared to standard brief nutritional guidance. We hypothesized that a group nutritional intervention would lead to significant improvements in anthropometric measures, body composition, dietary quality, and quality of life parameters in the intervention group of patients with overweight and obesity compared with patients who underwent brief individual nutritional counseling.

## 2 Methods

The institutional Helsinki Committee of the Tel Aviv Sourasky University Medical Center (0156–21-TLVMC) approved the study. The ethical review was approved by a full board Helsinki Committee. All participants signed informed consent prior to participation.

### 2.1 Study design and population

This randomized controlled trial was conducted between September 2021 and April 2023 at an MMT clinic at Tel Aviv Sourasky University Medical Center, Israel. The clinic is an ambulatory care service treating approximately 300 patients, aged ≥ 18 years who meet DSM-5 criteria for opioid use disorder. Inclusion criteria were patients with a BMI ≥ 25 kg/m², who were Hebrew-speaking with the cognitive ability to provide informed consent.

### 2.2 Randomization and intervention

Of the 300 patients who were in MMT at August 2021, those with a minimum of 6 months in MMT, with latest available BMI information BMI≥ 27 and or had high glycosylated hemoglobin ≥ 5.7 (n = 88) were asked to participate. Of them, (see flow diagram) a total of 77 patients who met the inclusion criteria were recruited and allocated to the intervention (n = 44) or control (n = 33) groups based on simple random method. Seven participants (two from the intervention and five from the control group) were excluded during the study: two due to deaths, one due to disciplinary removal, and four due to voluntary withdrawal, leaving 70 participants who completed the study.

Flow Diagram

**Figure:**
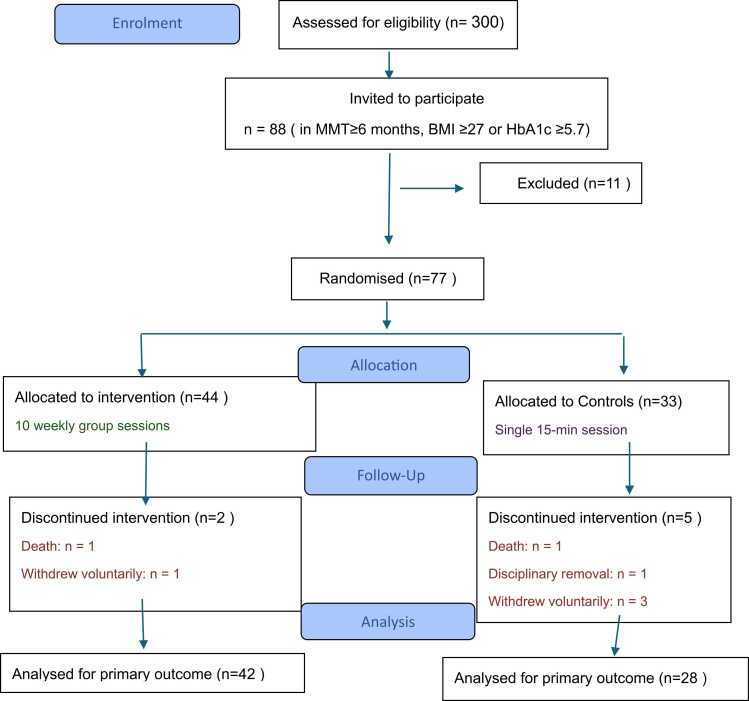

### 2.3 Intervention group

The intervention consisted of ten weekly group sessions (45 min each) led by a registered clinical dietitian. Groups included approximately 10 participants per session. The curriculum covered the following topics: Mediterranean nutrition principles; understanding food labeling; the health effects of sugar and processed food consumption; proper hydration habits; meal planning and organization; physical activity promotion; and self-control strategies for eating behaviors. Sessions utilized diverse educational materials including presentations, videos, and interactive activities. The final session included a group meal where participants prepared balanced, healthy plates using the knowledge gained throughout the program.

### 2.4 Control group

Control participants received a single 15-minute general nutrition guidance session at baseline, covering principles of healthy nutrition, encouragement of weight loss, and physical activity recommendations, along with written educational materials.

### 2.5 Measurements and assessments

All measurements were assessed twice, at baseline and at study completion by trained research staff.

### 2.6 Anthropometric measurements

Height and weight were measured using a calibrated electronic scale (XAVAX, model 113950, Germany) with a 100 g precision. BMI was calculated as weight (kg)/height (m²). Participants in the intervention group were weighed weekly during the sessions.

### 2.7 Body composition

Body composition was assessed using multifrequency bioelectrical impedance analysis (InBody S10, Korea) to determine lean body mass and body fat percentage. Measurements followed standardized protocols with participants fasting and adequately hydrated.

### 2.8 Dietary intake assessment

Dietary intake was evaluated using the validated Israeli Ministry of Health food frequency questionnaire (FFQ) (Shahar et al., 2003), administered through face-to-face interviews by trained dietitians. The FFQ assesses consumption frequency and portion sizes of 116 food items across various categories over the previous year.

### 2.9 Quality of life assessment

Quality of life was assessed using the Short Form 36 Health Survey (SF-36) (cHorney et al., 1994), a validated instrument covering eight domains: physical functioning, role limitations due to physical health, role limitations due to emotional problems, energy/fatigue, emotional well-being, social functioning, pain, and general health. Scores range from 0 to 100, with higher scores indicating better quality of life.

### 2.10 Demographic and clinical information

Demographic and clinical information were extracted from medical records, including substance use history, psychiatric medications, and methadone dosage.

### 2.11 Urine toxicology

Routine urine drug testing results (conducted twice monthly) were recorded for the one month prior to and during the study period. The urine samples were analyzed for opiates, cocaine metabolite (benzoylecgonine), benzodiazepines, and cannabis, using enzyme immunoassay systems (DRI®). The result for each drug was defined as positive if at least one urine test at each time point was positive.

### 2.12 Statistical analysis

Data analysis was performed using SPSS version 29. Continuous variables were compared using ANOVA and presented as means ± standard deviations. Categorical variables were compared using chi-square and Fisher's exact tests and presented as percentages. Statistical significance was set at p < 0.05. Changes in anthropometric and body composition measures, dietary intake and quality of life measures were analyzed using paired *t*-tests. Repeated measures ANOVA analysis was use to examine selected group effects (intervention vs. control; weight reduction vs. no weight reduction groups). Effect estimates (the mean difference (delta) (control minus intervention time) at baseline and post intervention, are presented with 95% confidence intervals (are presented in Table 2, Table 3, Table 4, Table 5).

**Table 1:** Baseline characteristics and demographics.

Characteristic	Intervention N(%) 42(100)	Control N(%) 28(100)	p value
**Sociodemographic**			
Male sex	31 (73.8)	22 (78.6)	0.8
Age, years*	53.9 ± 9.4	55.0 ± 8.5	0.6*
Israeli-born	27 (64.3)	18 (64.3)	1
Living with partner	16 (38.1)	11 (39.3)	1
Have children	33 (78.6)	22 (78.6)	1
Education ≥ 12 years	8 (19.0)	9 (32.1)	0.3
**Substance Use History**			
History of injection drug use	21 (50.0)	17 (60.7)	0.5
Substance use at recruitment	10 (23.8)	13 (46.4)	0.07
**Anthropometric Measures**			
Weight, kg*	92.1 ± 17.1	93.0 ± 17.3	0.9*
BMI, kg/m²*	32.0 ± 5.8	31.4 ± 4.3	0.6*
*BMI categories*			0.3
Overweight (25–29.9)	18 (42.9)	9 (32.1)	
Obesity Class I (30–34.9)	10 (23.8)	15 (53.6)	
Obesity Class II+ (≥35)	14 (33.3)	4 (14.3)	

Chi-square/Fisher's exact test * mean ± standard deviation for continuous variables, ANOVA.

BMI =  Body Mass Index.

**Table 2:** Changes in energy and macronutrient intake between groups at baseline and study completion.

	Intervention Group (n = 35)		Control Group (n = 25)		p Values			Delta, 95% CI Controls-intervention
	Baseline	Post	Baseline	Post	T	T*G	G	Baseline, and post
Energy Intake (kcal/day)	4416 ± 2377	3400 ± 1748	5506 ± 2614	3623 ± 1739	< 0.001	0.2	0.2	936, −396–2268 210.4, −735–1156.4
Protein (g/day)	144.1 ± 72.1	131.7 ± 55.2	200.7 ± 118.5	127.8 ± 65.3	< 0.001	0.01	0.2	51.1, 0.01–102 -7.7, −40.2–24.8
Total fat (g/day)	184.5 ± 111.6	144.2 ± 76.5	231.5 ± 123.9	148.4 ± 80.3	< 0.001	0.2	0.3	40.4, −22.6–103.5 3.2, −38.8–45.2
Cholesterol (mg/day)	599.7 ± 400.5	519.9 ± 284.1	778.2 ± 485.9	475.1 ± 283.2	< 0.001	0.036	0.6	163.6, −73.7–400 -63, −216.6–90.5
Saturated fat (g/day)	66.7 ± 44.2	56.4 ± 30.6	82.8 ± 47.1	54.6 ± 29.4	< 0.001	0.4	0.3	13.9, −10.7–38.6 5.3, −10.9–21.6
Carbohydrates (g/day)	531.9 ± 287.5	379.4 ± 222.8	642.0 ± 297.3	431.4 ± 213.4	< 0.001	0.5	0.3	92.7, −64–249 42.8, −76–161
Sugar (g/day)	273.0 ± 154.0	177.8 ± 112.6	320.5 ± 161.9	214.7 ± 120.6	< 0.001	0.9	0.3	38, −46.8–122.8 31.4, −31.9–94.8
Dietary fiber (g/day)	40.3 ± 24.7	38.3 ± 27.0	49.6 ± 25.2	34.5 ± 18.9	0.019	0.09	0.7	8.1, −5.4–21.6 -3.9, −16.9–9.2

Mean ± standard deviation are presented. Repeated measures multivariate analyses.

T- time, G- group, T*G - interaction between time and group

**Table 3:** Changes in calorie source category between Baseline and Post-Intervention by intervention and control groups.

	Intervention Group (n = 35)		Control Group (n = 25)		p Values			Delta, 95% CI Controls-intervention
	Baseline	Post	Baseline	Post	**T**	T*G	**G**	Baseline, and post
Carbohydrate	48.2 ± 6.6	44.7 ± 6.1	47.5 ± 6.8	48.4 ± 7.0	0.194	0.031	0.3	-0.7, −4.3–2.9 3.7, 0.25–7.2
Protein	13.8 ± 3.0	16.9 ± 3.5	14.7 ± 4.1	14.5 ± 3.1	0.003	< 0.001	0.4	0.18, −1.3–1.6 -3.1, −4.4—1.9
Fat	38.0 ± 5.4	38.4 ± 4.4	37.7 ± 4.1	37.0 ± 5.7	0.859	0.5	0.4	-0.22, −2.9–2.4 -1.3, −4.0–1.3

Mean ± standard deviation are presented. Repeated measures multivariate analyses. T- time, G- group, T*G - interaction between time and group.

**Table 4:** Changes in daily energy intake from selected processed food categories between baseline and post-intervention.

	Intervention Group (n = 35)		Control Group (n = 25)		p Values			Delta, 95% CI Controls-intervention
Food Category	Baseline	Post	Baseline	Post	**T**	T*G	**G**	Baseline, and post
Processed grains (kcal/day)	334.3 ± 283.1	233.6 ± 213.4	409.7 ± 361.7	348.8 ± 322.8	0.074	0.5	0.16	49.5, −77.6–176.6 102.3, −6.1–210.6
Sweet snacks (kcal/day)	1135.8 ± 996.8	633.3 ± 607.3	1215.6 ± 798.0	726.6 ± 673.0	< 0.001	0.8	0.7	36.6, −462.5–535.8 87.5, −259.7–434.7
Sweetened beverages (kcal/day)	277.1 ± 299.8	94.8 ± 198.2	331.4 ± 306.4	219.1 ± 198.6	< 0.001	0.4	0.19	38.7, −127–204 110.2, 2.2–218.3

Mean ± standard deviation are presented. Repeated measures multivariate analyses. T- time, G- group, T*G - interaction between time and group.

**Table 5:** Quality of life measures at baseline and post-intervention by group.

	Intervention Group (n = 38)		Control Group (n = 25)		p-Value			Delta, 95% CI Controls-intervention
SF-36 Domain	Baseline	Post	Baseline	Post	**T**	G*T	**G**	Baseline, and post
Physical Functioning	68.3 ± 29.2	69.7 ± 28.5	71.6 ± 24.6	62.0 ± 32.9	0.13	**0.045**	0.7	3.3, −10.8–17.4 -7.7, −23–7.9
Role Limitations - Physical	48.7 ± 45.4	45.4 ± 46.4	43.0 ± 44.8	39.0 ± 46.2	0.5	0.95	0.6	-5.7, −28.9–17.6 -6.4, −30.2–17.5
Role Limitations - Emotional	62.3 ± 46.6	50.0 ± 48.2	61.3 ± 47.8	38.6 ± 48.7	**0.025**	0.5	0.5	-0.94, −25.2–23.3 -11.3, −36.3–13.6
Energy/Vitality	47.9 ± 18.2	47.4 ± 15.6	43.4 ± 16.9	39.8 ± 23.5	0.4	0.6	0.1	-4.5, −13.6–4.6 -7.5, −17.4–2.3
Emotional Health	43.2 ± 13.0	42.0 ± 17.2	40.0 ± 13.9	37.5 ± 16.7	0.4	0. 8	0.2	2.5, −4.6–9.5 1.1, −4.5–6.8
Social Functioning	65.5 ± 28.5	67.1 ± 30.8	61.0 ± 31.1	52.5 ± 35.3	0.4	0.2	0.2	-4.5, −19.7–10.8 -14.6, −31.4–2.2
Bodily Pain	60.9 ± 32.3	59.7 ± 36.6	57.7 ± 35.4	55.4 ± 40.9	0.7	0.9	0.6	-3.2, −20.7–14.3 -4.3, −24.4–15.7
General Health	54.9 ± 22.6	50.0 ± 19.6	54.2 ± 25.1	48.4 ± 21.9	0.07	0.9	0.8	-0.7, −12.9–11.5 -1.6, −12.2–9.0

Mean ± standard deviation are presented. Repeated measures multivariate analyses. T- time, G- group, T*G - interaction between time and group.

## 3 Results

### 3.1 Participant characteristics

The 42 intervention and 28 control participants did not differ by sociodemographic characteristics, substance use history, or anthropometric measures (Table 1).

### 3.2 Body weight & body mass index

There was no significant difference in weight change between the intervention and control groups over the study period (time*group interaction p = 0.4). Mean weight remained stable in both groups, with the intervention group changing from 92.1 ± 17.1 kg to 92.0 ± 17.4 kg (change −0.1 kg) and the control group from 93.0 ± 17.3 kg to 92.0 ± 18.6 kg (change −1.0 kg).

Similarly, BMI of intervention changed from 32.0 ± 5.8–31.9 ± 5.7 kg/m² and controls from 31.4 ± 4.3–31.0 ± 4.7 kg/m²) (p(Time)= 0.2, p(Time*Group)= 0.4 and p(Group)= 0.5). Of all participants, 32 lost weight and 38 either maintained or gained weight, with no between-group difference (p = 0.6)

### 3.3 Body composition lean body mass

Lean body mass did not change over time (pT=0.7) and between groups (p = 0.9), with expected sex difference (pG<0.001). Specifically, in males, intervention group from 63.8 ± 13.3 kg to 62.3 ± 7.4 kg and control from 63.7 ± 11.5 kg to 64.6 ± 10.6 kg. Among females, the intervention group changed from 47.3 ± 7.1 kg to 49.2 ± 6.8 kg, and controls from 47.8 ± 6.7 kg to 48.3 ± 6.5 kg. Body fat percentage changed over time (pT=0.008) and between sexes (pG=0.025), with a trend toward significance interaction (pT*G=0.06). Specifically, among males, the control group showed a reduction from 32.6 ± 7.8% to 30.8 ± 7.5%, while the intervention group remained stable (35.3 ± 9.0% to 35.3 ± 8.4%). Among females, both groups demonstrated reductions: the intervention group from 39.8 ± 8.8% to 38.8 ± 9.3% and the control group from 41.3 ± 7.0% to 37.1 ± 7.5%.

### 3.4 Dietary composition

#### 3.4.1 Macronutrient and energy intake

As detailed in Table 2, both groups demonstrated significant reductions in total daily caloric intake (repeated-measures analysis, p(Time)< 0.001) with no other differences (p(Time*Group)= 0.3,p(Group)= 0.2). However, change in protein intake differed between groups (Repeated-measures analysis, p(Time)< 0.001, p(Time*Group)= 0.01, p(Group)= 0.2). Specifically, protein intake did not change in the intervention group (144.1 ± 72.1–131.7 ± 55.2 g/day) but decreased in the control group (200.7 ± 118.5–127.8 ± 65.3 g/day). Total fat intake and saturated fat reduced in both groups, with no time-by-group interaction effect (Table 2). Carbohydrate intake also decreased significantly in both groups, as did sugar consumption. With respect to dietary fiber intake, a significant reduction with a trend toward a significant interaction was found (Table 2). Paired *t*-tests revealed that the intervention group preserved dietary fiber intake (40.3 ± 24.7–38.3 ± 27 g/day, paired *t*-test p = 0.7), while the control group showed a decrease (49.6 ± 25.2–34.5 ± 18.9 g/day, paired *t*-test p = 0.002).

#### 3.4.2 Caloric source distribution

A change in caloric source distribution was observed in the intervention group (Table 3). With respect to carbohydrate intake, no change over time (p(Time)= 0.2), but an interaction effect (p(Time*Group)= 0.031) reflecting a reduction in the proportion of calories derived from carbohydrate in the intervention group (from 48.2 ± 6.6% to 44.7 ± 6.1%) but not in the controls (47.5 ± 6.8% to 48.4 ± 7.0%). Moreover, the proportion of calories derived from protein increased in the intervention group (13.8 ± 3.0% to 16.9 ± 3.5%) but not in the controls (14.7 ± 4.1% to 14.5 ± 3.1%) (p(T)= 0.003, p(Time*Group)< 0.001, p(G)= 0.4). Fat intake remained unchanged in both groups (Table 3).

#### 3.4.3 Ultra-processed food (UPF)

Analysis of percentage of daily calories from UPF revealed a reduction in UPF consumption in both groups (p(T)= 0.001), with no interaction or between group differences (p(T*G)= 0.5, p(G)= 0.2). Using paired *t*-tests, UPF intake decreased in the intervention group (from 42.1 ± 1.6% to 31.9 ± 1.7%, p = 0.001), but not in the controls (from 45.3 ± 1.6% to 38.7 ± 1.6%, p = 0.1).

When examining specific categories of processed foods, both groups demonstrated reductions in caloric intake from sweet snacks and in sweetened beverage consumption, with no differences between groups (Table 4).

#### 3.4.4 Water intake

An increase in daily water consumption was demonstrated, with no group differences (p(Time)= 0.002, p(Time*Group)= 0.7, p(Group)= 0.6). Using paired *t*-test, the increase was in the intervention group (from 681.6 ± 609.9–1034.2 ± 559.9 ml/day, p < 0.001) but not in the controls (662.1 ± 599.5–935 ± 650.5 ml/day p = 0.078).

### 3.5 Quality of life measures

**Fig. 1:**
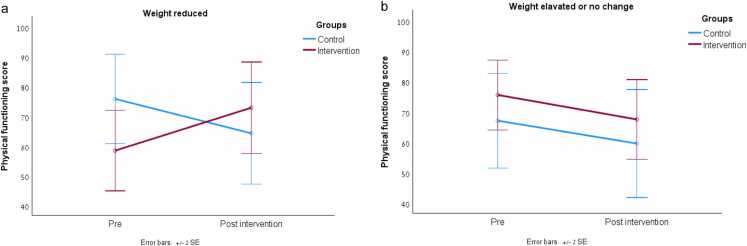
(a) Physical functioning score changes by study groups in patients who lost weight. (b) Physical functioning score changes by study groups in patients with weight gain or stable weight.

Quality of life domains were stable across most domains, except a decline in physical functioning observed in the controls group only (from 71.6 ± 24.6–62.0 ± 32.9, Time*Group interaction p = 0.045) (Table 5). Stratifying physical functioning by weight loss (n = 29) or weight gain/no change (n = 34) revealed differential effects within groups. While physical functioning scores deteriorated in controls who either gained weight or achieved weight loss, among the intervention group, score improved among those who successfully lost weight, but declined among those who did not (weight gain or no change) (Fig. 1a,b). Interaction effects p(Time*study groups)= 0.013, p(Time*weight groups)= 0.07, p(Time*study group*weight group)= 0.01.

Using paired *t*-tests, the emotional role limitations domain showed no changes in the intervention group but a decline in the controls (from 69.2 ± 48.0–28.2 ± 44.8, p = 0.011).

## 4 Discussion

This randomized controlled trial provides novel evidence that group nutritional therapy can meaningfully improve dietary quality among patients on MMT, specifically through a more favorable macronutrient distribution, a reduction in carbohydrate contribution and an increase in protein intake, and among those who achieved weight loss, an improvement in physical functioning. To our knowledge, this is the first study to evaluate the effects of a structured group nutritional intervention on dietary composition, body composition, and quality of life in this population. These findings are particularly timely given the absence of specific nutritional guidelines for patients on MMT and the well-documented elevated metabolic risk in this population (Vallecillo et al., 2018, Sason et al., 2024).

Study participants exhibited a high mean BMI (>30), consistent with the elevated rates of overweight and obesity documented in this population (Vallecillo et al., 2018, Peles et al., 2016). Although both groups demonstrated significant reductions in daily caloric intake, this did not translate into weight loss, a pattern consistent with our previous randomized trial in patients on MMT, in which a nutrition education intervention similarly improved nutritional knowledge and eating habits without producing changes in BMI (Sason et al., 2018). The absence of weight loss despite improved dietary quality may reflect the relatively short duration of both interventions rather than a fundamental limitation of nutritional therapy in this population. Longer interventions may be necessary to translate improvements in dietary quality into measurable anthropometric changes, particularly given the complex pharmacological and metabolic barriers characteristic of patients on MMT, as discussed below. Several biological and behavioral mechanisms may explain this dissociation between caloric reduction and weight stability. At the neurobiological level, methadone's agonist activity at μ-opioid receptors sustains hedonic eating drive and sweet food preference even in the context of improved dietary awareness (Mysels and Sullivan, 2010, Zador et al., 1996), potentially creating a ceiling effect on achievable dietary change. Methadone-associated insulin resistance may further impair the metabolic response to caloric reduction (Daniels et al., 2018, Cheng et al., 2013). Beyond pharmacological factors, psychiatric medications commonly used in this population are known to promote weight gain and may have counteracted dietary improvements (Schlienz et al., 2018). Physical activity, a key determinant of energy balance, was neither formally assessed nor incorporated as a structured intervention component. Given that patients on MMT are characterized by markedly low physical activity levels (Caviness et al., 2013), its absence likely constrained weight loss outcomes. These unmeasured factors represent limitations of the current study; however, as the randomized design ensures balanced distribution of such factors across groups in expectation, their primary effect is likely an attenuation of overall weight loss rather than a differential bias favoring the intervention group.

Regarding body composition, body fat percentage at baseline was consistent with BMI-based obesity classification, in line with previous research in patients on MMT (Sadek et al., 2016). A modest but statistically significant reduction in body fat percentage was observed across the entire cohort over time, with no significant between-group difference. This overall reduction may reflect the general decrease in caloric intake and improvement in dietary quality observed in both groups, including reduced sugar and UPF consumption, which may have modestly shifted energy balance even in the absence of measurable weight change. Notably, reductions in visceral and ectopic fat depots may occur disproportionately to changes in total body weight following dietary improvement, suggesting that body weight alone may underestimate the true cardiometabolic benefit of the intervention (Gepner et al., 2018). Lean body mass remained stable in both groups, consistent with the preservation of protein intake observed in the intervention group and the absence of structured resistance training across the study period.

The absence of significant between-group differences in body composition changes is consistent with the broader pattern observed in this study, namely, that the intervention produced meaningful improvements in dietary quality without translating into differential anthropometric or body composition outcomes within the study timeframe. In the absence of a significant caloric deficit or structured resistance exercise program, major alterations in body composition are unlikely to occur over short intervention periods (Lin et al., 2021, Muller et al., 2012). Longer intervention periods may therefore be required to detect such changes, particularly in populations with complex metabolic profiles including insulin resistance and high baseline adiposity (Holmes and Racette 2021).

Daily energy intake at baseline was exceptionally high, reflecting substantial consumption of sugar and UPF, a pattern consistent with the nutritional profile documented in patients undergoing opioid replacement therapy (Waddington et al., 2023). Both groups demonstrated significant reductions in total energy intake, carbohydrate, and fat consumption over time, with no significant between-group interaction for these macronutrients. These reductions may contribute to improved metabolic health through reduced glycemic load and decreased saturated fat exposure, potentially lowering cardiovascular risk and improving insulin sensitivity (Tricò et al., 2018, Chiavaroli et al., 2021).

Baseline sugar intake substantially exceeded WHO recommendations of 5–10% of total caloric intake (Gillon-Keren and Eilat-Adar 2022), consistent with the well-documented preference for sweet foods in this population. Both groups achieved significant reductions in sugar consumption, with the intervention group demonstrating a notably greater reduction in sugar-sweetened beverages compared to controls. This is particularly noteworthy given that μ-opioid receptor activation sustains hedonic drive toward sweet food consumption in patients on MMT (Mysels and Sullivan, 2010, Kolarzyk et al., 2005, Green et al., 2013), making meaningful reductions in sugar intake especially challenging to achieve in this population. The general improvement observed in both groups may be partially attributable to increased dietary awareness resulting from study participation (Hawthorne effect) (McCambridge et al., 2014), as well as the brief nutritional guidance provided to controls.

Despite the overall reduction in caloric intake, the intervention group successfully maintained dietary fiber consumption, whereas controls showed a pronounced decrease. This differential response likely reflects the intervention's emphasis on Mediterranean dietary principles, which prioritize fiber-rich foods such as vegetables, legumes, and whole grains. Preservation of dietary fiber is particularly clinically relevant in patients on MMT, who commonly experience opioid-induced constipation and associated metabolic complications (Vallecillo et al., 2018).

Similarly, while the control group experienced a substantial reduction in absolute protein intake, the intervention group not only maintained stable protein consumption but also increased the proportional contribution of protein to total caloric intake. This macronutrient optimization, achieved through structured nutritional education rather than spontaneous dietary change, is clinically meaningful, as adequate protein intake supports lean body mass preservation and metabolic function during caloric restriction (Ogilvie et al., 2022). Furthermore, while both groups reduced total fat intake, the intervention group uniquely maintained stable monounsaturated fat consumption, a key component of the Mediterranean diet associated with cardiovascular benefit (Guasch-Ferre and Willett 2021), while the control group showed significant decreases. Taken together, these findings suggest that the group nutritional intervention produced a qualitatively superior dietary pattern, not merely a reduction in overall intake, compared to brief individual guidance.

Both groups demonstrated significant reductions in UPF consumption, which may be partially attributed to increased dietary awareness resulting from study participation (Hawthorne effect) (McCambridge et al., 2014) or the brief nutritional guidance provided to controls. Nevertheless, the intervention group achieved a substantially greater reduction, bringing its UPF consumption below general Israeli population levels, whereas the control group remained above these levels (Gillon-Keren and Eilat-Adar 2022). This differential response likely reflects the intervention's explicit focus on UPF recognition and food label literacy, skills that equip participants to make informed food choices independently of professional guidance.

The clinical significance of this finding extends beyond caloric reduction. UPF are characterized by high palatability and are specifically engineered to activate reward pathways, making them particularly difficult to avoid for individuals with dysregulated reward systems such as patients on MMT (Contreras-Rodriguez et al., 2022, Hough et al., 2026). Reducing UPF consumption in this population is therefore especially meaningful given the high prevalence of metabolic syndrome (54%) among patients on MMT, substantially exceeding rates in the general population (Vallecillo et al., 2018), and their elevated cardiovascular risk (Sason et al., 2024, Holmes and Racette, 2021).Daily water consumption increased in both groups over time, with a more pronounced improvement in the intervention group, reflecting the effectiveness of the dedicated hydration education component, including the substitution of caloric, sugar-sweetened beverages with water. The improvement observed in the control group likely reflects increased dietary awareness from study participation, as discussed above.

The health implications of improved hydration in this population are considerable. Substitution of sugar-sweetened beverages with water has been associated with meaningful reductions in cardiovascular-metabolic risk (Enhörning et al., 2019), with high sugar-sweetened beverage consumption linked to non-alcoholic fatty liver disease, insulin resistance, and hypertriglyceridemia through excess fructose metabolism and hepatic de novo lipogenesis (Malik and Hu, 2022) - cardiometabolic complications that are highly prevalent among patients on MMT (Vallecillo et al., 2018, Daniels et al., 2018). Beyond direct caloric reduction, adequate water intake may lower copeptin levels, a surrogate marker of vasopressin activity, thereby improving glucose homeostasis, particularly among individuals with habitually low water intake (Hakam et al., 2024, Chen et al., 2024). Given the high prevalence of insulin resistance and metabolic syndrome among patients on MMT, these metabolic benefits of improved hydration are especially clinically relevant in this population.

Baseline quality-of-life scores were markedly lower than general population norms, consistent with the well-documented impairment in physical and mental health domains among patients on MMT (De Maeyer et al., 2010). Despite the significant improvements in dietary quality achieved by the intervention group, overall quality-of-life scores remained stable, with no significant between-group differences, a finding that may reflect the relatively short intervention duration and the complex, multifactorial determinants of quality of life in this population.

The most clinically meaningful quality-of-life finding was the differential pattern in physical functioning. The intervention group maintained physical functioning scores over the study period, whereas the control group showed a significant decline. This protective effect of the intervention on physical functioning may reflect the combined benefits of improved dietary quality, reduced UPF consumption, and increased hydration, changes that collectively support metabolic health and physical capacity. Indeed, higher diet quality has been prospectively associated with a significantly lower risk of physical function impairment, as measured by the SF-36, in large cohort studies (Hagan et al., 2016).

When stratified by weight change, a more nuanced picture emerged. Among intervention group participants who achieved weight loss, physical functioning scores improved significantly, while those who did not lose weight showed a decline. In contrast, control participants experienced declines in physical functioning regardless of weight change, including those who lost weight, suggesting that weight reduction in the control group may have reflected unintentional loss associated with deteriorating health status rather than volitional dietary change.

The current study has limitations. The key limitations include the small sample size (n = 70) which is underpowered to find weight differences. In addition, the relatively short intervention duration (10 weeks), particularly for a population with complex metabolic and behavioral profiles, and may have been insufficient to produce measurable changes in weight or body composition. Additional limitations include the lack of blinding which may introduce performance bias, reliance on self-reported dietary and quality of life assessments. As we did a controlled clinical trial, in expectation, some of the unmeasured confounding should be ignorable. Still, lack of data on methadone treatment profiles, psychiatric medication use, and physical activity levels may have differentially influenced outcomes.

Future studies should employ longer intervention periods (e.g., 6 months with follow-up), larger sample sizes (n > 200 for adequate statistical power), and objective measures such as accelerometers for physical activity and DXA for body composition. Bundled approaches combining nutrition, exercise, and behavioral therapy may yield more robust anthropometric outcomes. Additionally, subgroup analyses by sex and methadone treatment duration, as well as cost-benefit analyses of group nutritional therapy in MMT settings, would further inform clinical implementation.

## 5 Conclusions

This randomized controlled trial demonstrates that group nutritional therapy can produce meaningful improvements in dietary quality among patients on MMT, including a more favorable macronutrient distribution, reduced ultra-processed food consumption, and improved hydration, changes with important cardiometabolic implications in this high-risk population. Among participants who achieved weight loss, quality of life as measured by physical functioning also improved. The improvements observed in both groups suggest that even brief nutritional guidance, combined with increased dietary awareness from study participation, can contribute to beneficial dietary changes in this population. Given the absence of specific nutritional guidelines for patients on MMT and their elevated risk for metabolic disorders, these findings support the integration of structured nutritional therapy into MMT programs.

## CRediT authorship contribution statement

**Ronit Anbar:** Visualization, Conceptualization. **Anat Sason:** Validation, Data curation. **Dror Ben Noach:** Writing – original draft, Project administration, Data curation. **Limor Ben Haim:** Conceptualization. **Einat Peles:** Writing – review & editing, Formal analysis, Conceptualization. **Miriam Adelson:** Visualization.

## Funding

Dr. Miriam and Sheldon G. Adelson Foundation for the Biology of Addictive Diseases; National Authority for Community Safety.

## Declaration of Competing Interest

All authors (Dror Ben Noach, Anat Sason, Ronit Anbar, Limor Ben Haim, Miriam Adelson, Einat Peles) declare that they have no known competing financial interests or personal relationships that could have appeared to influence the work reported in this paper.
